# Harnessing Telemedicine for the Provision of Health Care: Bibliometric and Scientometric Analysis

**DOI:** 10.2196/18835

**Published:** 2020-10-02

**Authors:** Ahmed Waqas, Soo Huat Teoh, Luís Velez Lapão, Luiz Ary Messina, Jorge César Correia

**Affiliations:** 1 Institute of Population Health University of Liverpool Liverpool United Kingdom; 2 Lifestyle Science Cluster Advanced Medical and Dental Institute Universiti Sains Malaysia Penang Malaysia; 3 Global Health and Tropical Medicine Instituto de Higiene e Medicina Tropical Universidade Nova de Lisboa Lisbon Portugal; 4 Telemedicine University Network Rede Nacional de Ensino e Pesquisa Brasília Brazil; 5 Unit of Patient Education Division of Endocrinology, Diabetology, Nutrition and Patient Education Department of Medicine, Geneva University Hospitals and University of Geneva Geneva Switzerland

**Keywords:** telemedicine, scientometric analysis, evidence synthesis, health information technology, research, theme

## Abstract

**Background:**

In recent decades, advances in information technology have given new momentum to telemedicine research. These advances in telemedicine range from individual to population levels, allowing the exchange of patient information for diagnosis and management of health problems, primary care prevention, and education of physicians via distance learning.

**Objective:**

This scientometric investigation aims to examine collaborative research networks, dominant research themes and disciplines, and seminal research studies that have contributed most to the field of telemedicine. This information is vital for scientists, institutions, and policy stakeholders to evaluate research areas where more infrastructural or scholarly contributions are required.

**Methods:**

For analyses, we used CiteSpace (version 4.0 R5; Drexel University), which is a Java-based software that allows scientometric analysis, especially visualization of collaborative networks and research themes in a specific field.

**Results:**

We found that scholarly activity has experienced a significant increase in the last decade. Most important works were conducted by institutions located in high-income countries. A discipline-specific shift from radiology to telestroke, teledermatology, telepsychiatry, and primary care was observed. The most important innovations that yielded a collaborative influence were reported in the following medical disciplines, in descending order: public environmental and occupational health, psychiatry, pediatrics, health policy and services, nursing, rehabilitation, radiology, pharmacology, surgery, respiratory medicine, neurosciences, obstetrics, and geriatrics.

**Conclusions:**

Despite a continuous rise in scholarly activity in telemedicine, we noticed several gaps in the literature. For instance, all the primary and secondary research central to telemedicine was conducted in the context of high-income countries, including the evidence synthesis approaches that pertained to implementation aspects of telemedicine. Furthermore, the research landscape and implementation of telemedicine infrastructure are expected to see exponential progress during and after the COVID-19 era.

## Introduction

Advances in information and communication technologies (ICTs) have virtually reduced the world to a global village. The recent progress in ICTs has also shown incredible promise in addressing significant challenges in health care in disparate regions worldwide. Specifically, telemedicine ensures the provision of accessible, cost-effective, and specialized health care services in disparate areas. According to the World Health Organization (WHO), telemedicine pertains to the delivery of health care using different modalities embedded in the realms of information and communication technologies. It aims to advance health care, ranging from individual to population levels, by allowing exchange of patient information for diagnosis and management of health problems, primary care prevention, and education of physicians via distance learning [1]. Telemedicine is a new channel for health care services, which also enables opportunities to strengthen collaborative research.

The earliest evidence for telemedicine can be traced back to a clinical report published in *The Lancet* in 1879, which described the successful diagnosis of a child over the phone [2]. In addition, use of telegraphs was also evident in the American Civil War for transfer of mortality data and remote delivery of medical care [3]. A fine example of telemedicine was seen when, 20 years ago, the National Aeronautics and Space Administration monitored the astronauts’ well-being during the Apollo mission to the moon. The modern form of telemedicine, however, appeared with the advent and maturation of the internet, which made possible the use of videoconferencing, high-quality data transfer, and distance learning platforms at a lower cost. The potential of telemedicine in strengthening health systems was also recently recognized by the WHO, leading to the establishment of the Global Observatory for eHealth in 2005 [1]. In 2009, the telemedicine module of the Global Observatory for eHealth mapped the development of telemedicine in 4 specialties of medicine—pathology, radiology, psychology, and dermatology—in 114 member states. This report found that the greatest development had been made in the provision of teleradiology services (33%) among the WHO member states, while 20% of the countries reported conducting a national review or evaluation of telemedicine. In addition, 50% of the member states reported that they had institutions dedicated to the development of telemedicine solutions [1].

The most recent report by the WHO, published in 2016, emphasized the role of eHealth in achieving universal health coverage [4]. The development of telemedicine was found crucial in the attainment of sustainable development goal 8, “achieve universal health coverage,” and goal 3, “ensure healthy lives and promote well-being for all at all ages,” thus ensuring health for all, regardless of creed, ethnicity, color, or finances. It also identified a rapid progress among its member states from 2010 to 2016. At least 83% of the countries had reported a mobile health (mHealth) initiative, widespread use of teleradiology (from 33% to 77%), telepathology or teledermatology (about 50%), and telepsychiatry (33%). In addition, e-learning initiatives were reported in 84% of countries and the use of national electronic health records in 47% of the member states, and 78% of the countries reported legislations ensuring privacy of the electronic data. However, similar to the previous survey, very few countries had conducted evaluations of mHealth programs, which limits our understanding of the use of telemedicine, its barriers and facilitators, and which elements actually work [4]. These reports are a milestone in the field of telemedicine. However, these were dependent on data provided by government organizations and were heavily focused on government-run telehealth initiatives [4].

While the WHO-commissioned reports and evidence synthesis publications were indispensable in gauging worldwide infrastructure and legislation in telemedicine, scholarly research is a true marker for progress and evolution in every scientific field, and it is crucial to map research output in the field of telemedicine to determine prevalent research themes in order to guide policy makers and funders to improve or restrict flow of funding when required. Recognizing the importance of mapping progress in a field, scientists have devised several reproducible statistical methods that form the disciplines of bibliometrics and scientometrics. Scientometrics is defined as the “quantitative study of science, communication in science, and science policy” [5], and it helps evaluate the impact of journals, scientists, and institutions on the development and innovation of a scientific field.

Several studies published recently have used bibliometric methods to study progress in telemedicine, albeit in a very narrow context. For instance, Fatehi and Wootton [6] focused on delineating the use of different terminology to describe telemedicine, Groneberg et al [7] detailed the country-specific publication output and annual trends of publication and citation outputs, Gu et al [8] described the intellectual structure of telemedicine research by focusing on collaborative networks between different countries and authors, and Askari et al [9] provided an overview of the top 60 most frequently cited studies in telemedicine-specific journals. There is, however, a paucity of studies providing a holistic snapshot of advances in telemedicine from 2010 to 2019. The present analysis leverages the use of scientometric techniques to analyze publication output in the field of telemedicine worldwide, regardless of the government, industrial, or academic affiliations.

## Methods

### Bibliographic Search

An academic search of the Web of Science (WOS) core database was performed covering January 2010 to December 2019 to retrieve English language papers, using the following keyword: TS=(telemedicine). We restricted our search results to papers published in English only. The bibliographic records of these studies, including titles, abstracts, author details, affiliations, keywords, and citing references, were downloaded. We restricted our search results to the last 10 years to restrict our analyses in order to achieve a snapshot of recent research activity in telemedicine. The WOS core database was used for this academic search for two important reasons: it provides large coverage of over 20,000 peer-reviewed journals pertaining to 250 disciplines in health and social sciences and engineering and it is the only database that allows curation of citing references of each indexed publication to allow cocitation analyses.

### Operational Definitions

Telemedicine literally means “healing at a distance.” However, there are no definitive definitions of telemedicine, with a recent review reporting over 104 peer-reviewed definitions found in the literature [10]. However, for the purpose of this scientometric investigation, we adapted a definition of telemedicine embodying 4 crucial elements: (1) provision of clinical support, (2) connection of users from different physical locations, (3) improved health outcomes, and (4) use of ICT [4,6].

### Knowledge Visualization Analyses

For analyses, we used CiteSpace (version 4.0 R5; Drexel University), which is a Java-based software that allows scientometric analysis, especially visualization of collaborative networks and research themes in a specific field [11,12]. The visualization of these collaborative networks in a discipline is based on the theory of cocitation, which posits that 2 documents share a cocitation relationship when they are cited together by another document [11,12]. For mapping of these networks, we ran network analyses using the cosine link reduction method and pathfinder networking scaling. Term sources were set as titles, abstracts, and author keywords, while nodes were set as cited references to delineate collaborative networks and cluster analyses. The time-splicing method was used to explore publications in 2 periods, 2010 to 2014 and 2015 to 2019, where each slice comprised the top 50 cited papers every year.

To obtain clusters or themes of research, we ran cluster analyses in which each cluster was termed according to publication keywords using 2 text-mining methods: term frequency-inverse document frequency (TF-IDF) and log likelihood ratio (LLR). The first method, TF-IDF, uses terms that are weighted by term frequencies multiplied by inverted document frequencies [11,12]. LLR tests choose the most appropriate clustering label by assessing the strength of the bond between a term and a cluster [11,12]. A cluster is said to be parsimonious when it comprises a larger number of items and an acceptable silhouette and modularity value (Q). The silhouette value is a measure of how similar an object is to its own cluster (cohesion) compared with other clusters (separation) [11,12]. The value of Q and silhouette ranges between –1 to 1, where a value closer to 1 is considered acceptable.

Each paper is presented as a node and links between two nodes as edges. The collaborative network mapped from this analysis yields several important results. Any research study with centrality values ≥0.1 are considered influential in their collaborative networks. Citation rings show annual citation patterns, while purple nodes represent landmark theories or works that give rise to a new body of work. Citation bursts revealing short periods of high scholarly activity are presented as red rings. Based on these cues, researchers can identify important works in a field and important themes of research.

## Results

### Bibliometrics

**Figure 1:**
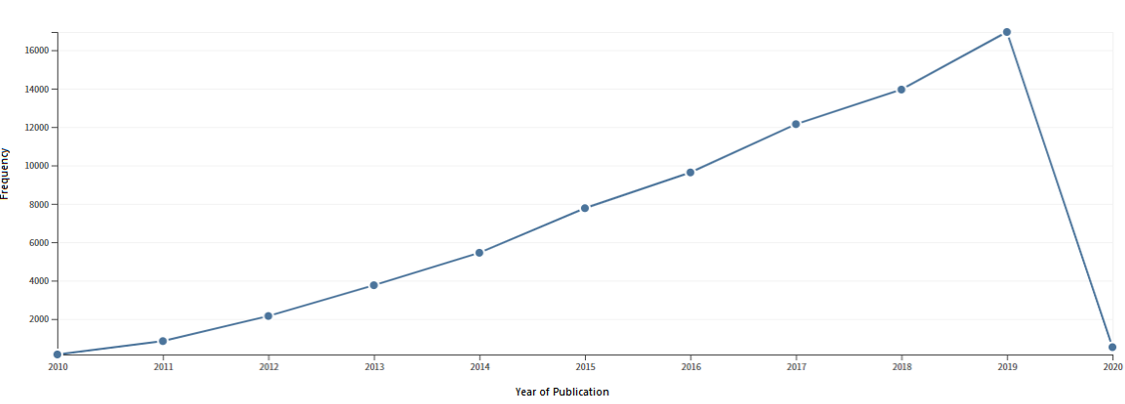
Trends of citations received by papers in telemedicine.

Web of Science (core database) yielded 6896 publications from 2010 to 2019, with a total h-index of 87 and an average 10.64 citations per study. These were cited a total of 73,354 times by a total of 42,381 citing papers. There was an increasing trend in both the publication and citation output from 2010 to 2019 (Figure 1).

### Regional Trends in Telemedicine Research

**Figure 2:**
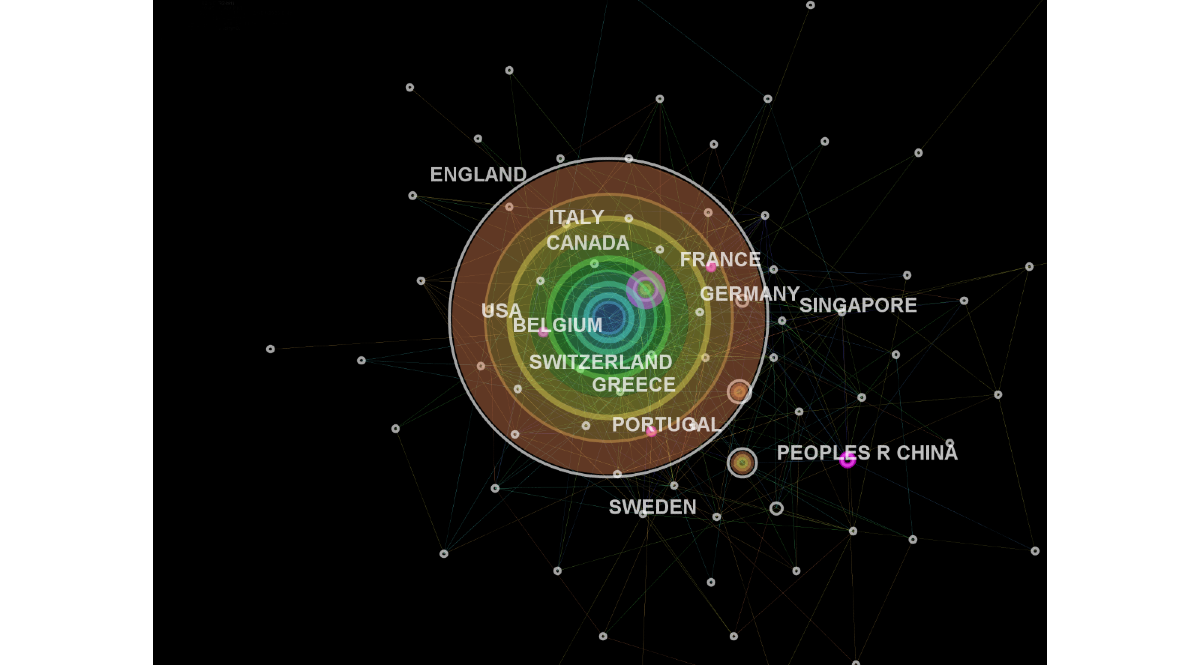
Regional collaborative networks in the area of telemedicine research. Peoples R China: People’s Republic of China; USA: United States of America.

Regionally, the highest publication output (in English) was contributed by high-income countries: the United States, Australia, England, Canada, and Germany. There were 2 middle-income countries, India and the People’s Republic of China, that also ranked in the top 10 for publication output. However, in terms of having a centrality score of 0.1 or greater, 6 countries—England, France, Belgium, Portugal, the People’s Republic of China, and Greece—appeared to hold significant influence in worldwide collaborations in telemedicine. These countries are also presented as purple nodes in Figure 2 (ie, contributing groundbreaking research). These central nations, although mainly high-income countries, also formed collaborations with a number of low- and middle-income countries (LMICs), such as Ethiopia, Mali, Botswana, Nepal, Zimbabwe, Pakistan, and Uganda.

### Institutional Trends in Telemedicine Research

**Figure 3:**
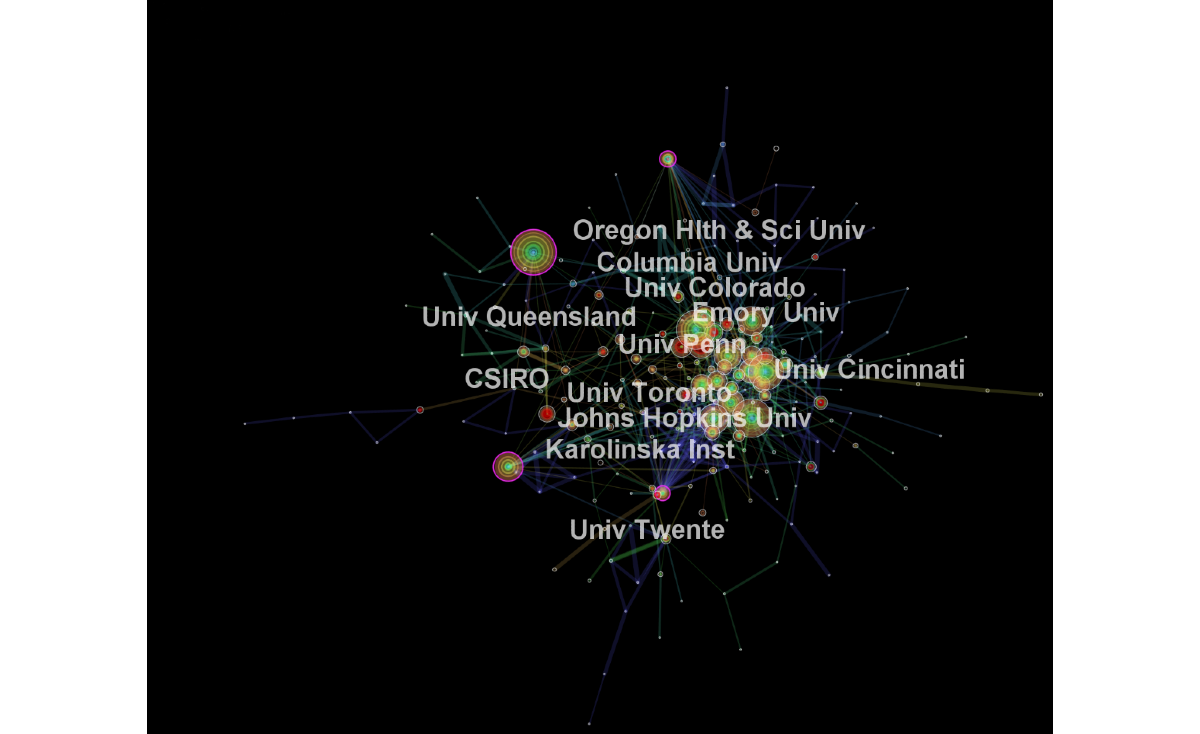
Institutional collaborations in telemedicine research. CSIRO: Commonwealth Scientific and Industrial Research Organisation; Hlth: Health; Inst: Institute; Sci: Science; Univ: University.

Among institutions, the top 9 contributing institutions in terms of publication output were based in the United States, including the University of California system (n=304), Harvard University (n=227), and the Pennsylvania Commonwealth System of Higher Education (n=152). Outside of the United States, the University of Queensland in Australia was the fifth-highest contributing region. Institutions with centrality ≥0.1 included Columbia University, University of Queensland, University of Toronto, and Karolinska Institute. It is noteworthy that none of the top 9 US institutions in terms of publication output were deemed important in their collaborative networks (Figure 3).

### Discipline-Specific Trends in Telemedicine Research

**Figure 4:**
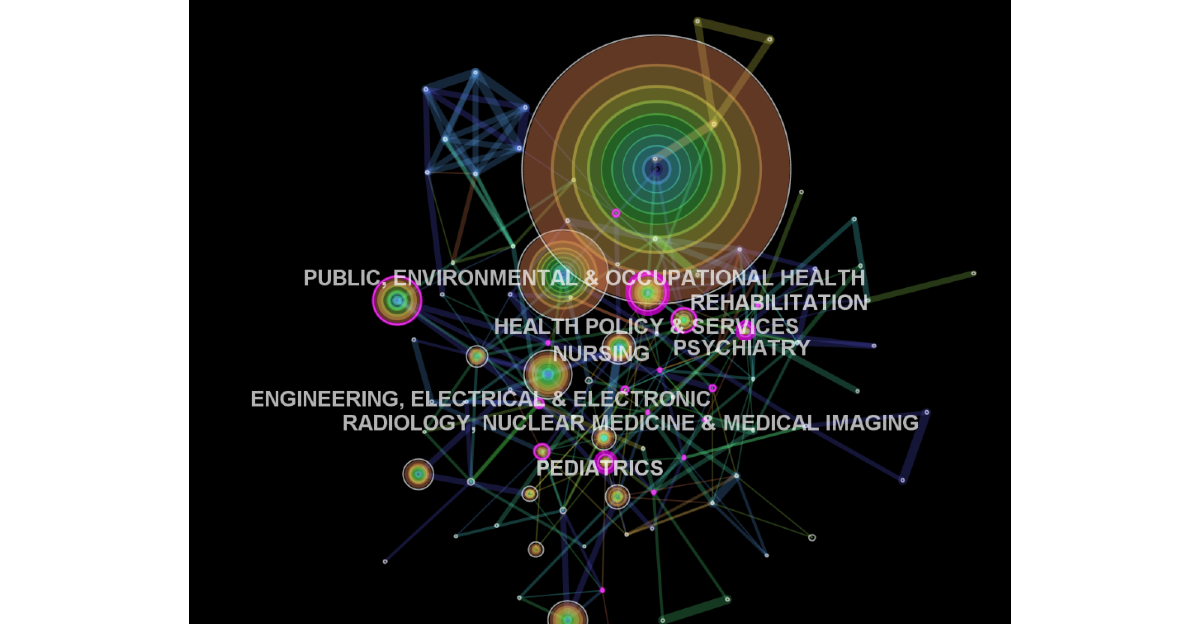
Discipline-specific trends in telemedicine research.

An analysis of the Web of Science revealed that the disciplines of health care sciences and services (n=2338) and medical informatics (n=835) reported the highest number of publication items. However, a total of 19 disciplines had the greatest centrality values (≥0.1). Most important innovations yielding a collaborative influence were reported in the following medical disciplines, in descending order: public environmental and occupational health, psychiatry, pediatrics, health policy and services, nursing, rehabilitation, radiology, pharmacology, surgery, respiratory medicine, neurosciences, obstetrics, and geriatrics (Figure 4).

### Keyword Analysis in Telemedicine

**Figure 5:**
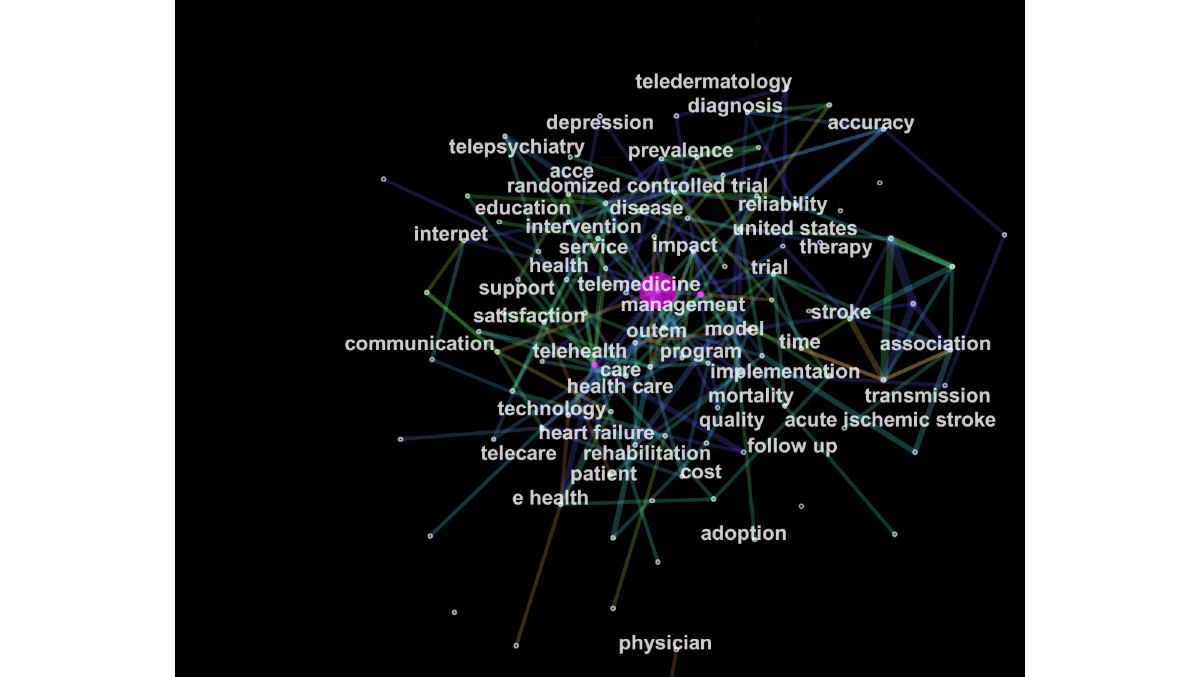
Research keywords in telemedicine research. acce: access; outcm: outcome.

**Figure 6:**
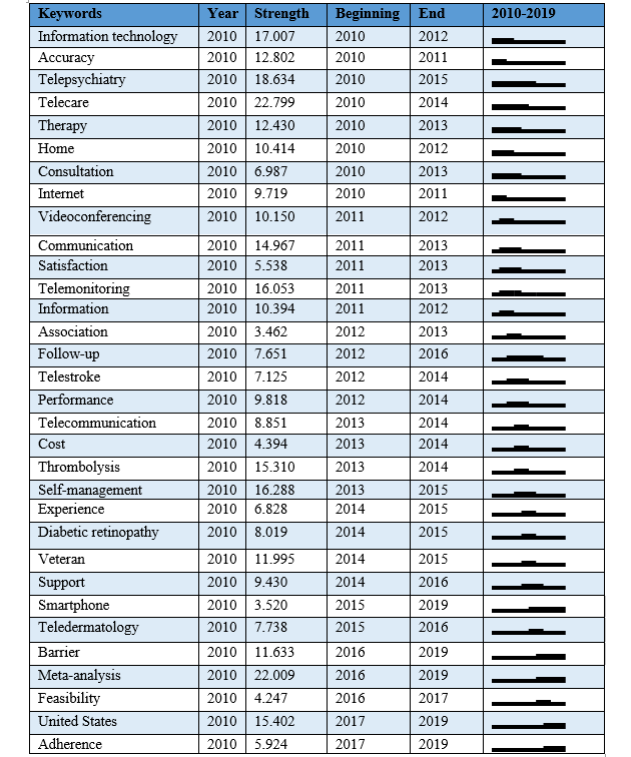
Research hotspots in telemedicine from 2010 to 2019.

A total of 106 research keywords were identified in the field of telemedicine, revealing the most-researched topics (Figure 5). From 2010 to 2019, 32 keywords appeared to have citation outbursts showing the greatest research activity in telemedicine (Figure 6). The top 50 cited keywords in tandem with citation outbursts were divided into themes to identify the most frequently researched diseases, outcomes, study designs, and populations, shown in Table 1.

**Table 1:** Research keywords in telemedicine by theme.

Theme	Most frequent keywords
Diseases	Diabetic retinopathy, stroke, depression, heart failure, rehabilitation, thrombolysis
Performance indicators	Feasibility, accuracy, reliability, barrier, adherence, satisfaction, performance, cost-effectiveness
Outcomes	Self-management, support, impact, diagnosis, education, mortality, quality of life, telemonitoring
Study design	Association, follow-up, meta-analysis, randomized controlled trials, implementation, prevalence
Disciplines	Telestroke, teledermatology, telepsychiatry, primary care
Setting	Home, internet, videoconferencing, communication, telecommunication, smartphone
Population	Veterans, United States, children

### Clusters of Research in Telemedicine From 2009 to 2014

**Figure 7:**
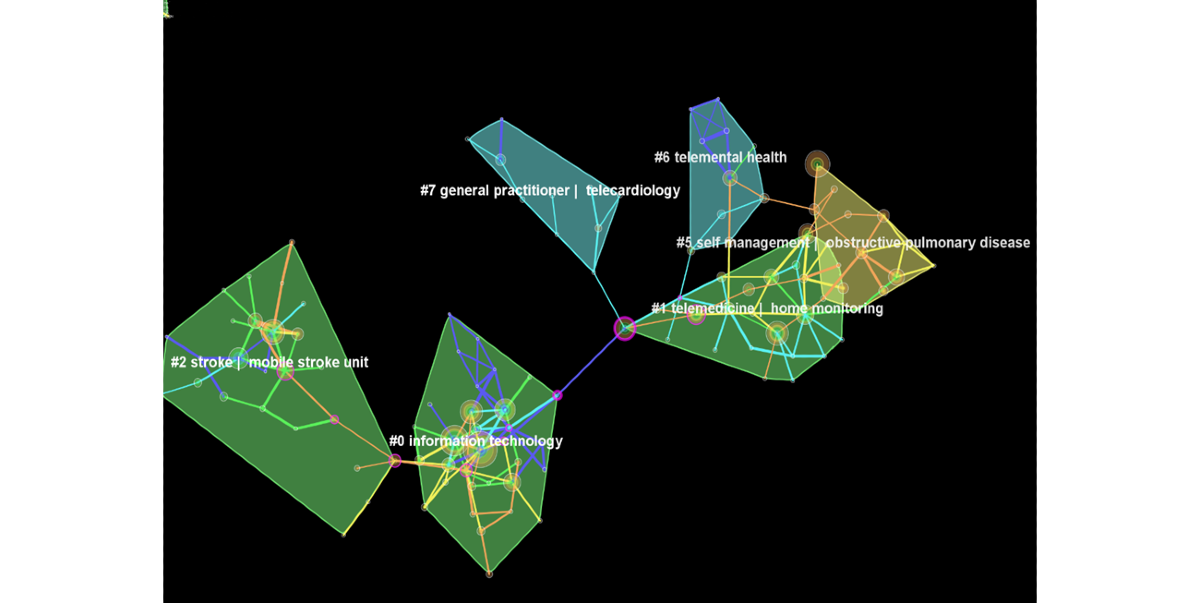
Clusters of research in telemedicine from 2009 to 2014.

From 2009 to 2014, a total of 2527 papers were published, which were cited by 141,702 references. These were analyzed to study landmark publications and clusters of research during this period. There was a total of 228 nodes and 273 edges. Cluster analysis yielded a parsimonious network of clusters (Figure 7) with a modularity of 0.85 and silhouette value of 0.42. It yielded a total of 56 clusters, out of which 8 comprised at least 10 studies each and an acceptable silhouette value (range 0.85 to 1.0).

#### Acute Medicine

##### Stroke

The zeroth cluster comprised 32 items with a silhouette value of 0.98, described by terms such as information technology (TF-IDF) and acute ischemic stroke and thrombolysis (LLR). The third cluster pertained to mobile stroke units (TF-IDF) and associated mortality and economic outcomes (LLR).

##### Telecardiology

The second cluster pertained to telemonitoring at home (TF-IDF) for chronic diseases such as heart failure (LLR). The fifth cluster was focused on clinical management and home monitoring (TF-IDF) of pacemaker activity and implantable cardioverter defibrillator (LLR), especially in patients with myocardial infarction.

#### Chronic Diseases

##### Diabetes

Cluster 7 pertained to general practitioner–mediated telecardiology (TF-IDF) and user acceptance of this program (LLR). The sixth cluster pertained to nutrition in diabetes, especially in the context of the Columbia University Informatics for Diabetes Education and Telemedicine project.

##### Telepsychiatry

The third cluster was defined as neuropsychological assessment (by TF-IDF method), focusing on posttraumatic stress disorder (PTSD) and cognitive behavioral therapy (LLR method).

##### Respiratory Medicine

This cluster (the fifth cluster) pertained to self-management of chronic obstructive pulmonary disease (COPD) and its exacerbation (TF-IDF and LLR).

### Landmark Publications From 2009 to 2014

The period from 2009 to 2014 revealed 10 landmark publications pertaining to different themes, where most of the publications pertained to intensive care, especially stroke (Table 2) [13-16]. Audebert et al published 3 important studies [17-19] demonstrating the success of the telemanagement of stroke in rural hospitals in Bavaria, Germany. In a similar context, Schwamm et al [13] provided evidence for telestroke consultations via videoconferencing and Lilly et al [14] showed that the implementation of a tele–intensive care unit (ICU) intervention was associated with reduced adjusted odds of mortality and reduced length of hospital stay, as well as with changes in best-practice adherence and lower rates of preventable complications. In their cross-sectional survey, Silva et al [16] described the status of telestroke programs in the United States. In addition, 2 important literature reviews were published during this period. Sood et al [10] improved our understanding of the theoretical underpinnings of modern telemedicine after a careful evaluation of 104 peer-reviewed publications, while Kahn et al [15] summarized the recommendations of a working group for the adoption of a standardized framework for the standardized conduct of tele-ICU studies. Lastly, May et al [20] described the complexity that exists in the scale-up of telemedicine programs, which is often underestimated, leading to their failures.

**Table 2:** Lessons learned from landmark publications from 2009 to 2014.

Author (year)	Study design	Theme	Disease	Lesson learned
Broens (2007) [21]^a^	Qualitative literature review	Implementation	N/A^b^	Determinants of successful implementation and scale-up of telemedicine programs. Important determinants of telemedicine programs include (1) technology, (2) acceptance, (3) financing, (4) organization, and (5) policy and legislation.
Schwamm et al (2004) [13]	Retrospective	Feasibility	Stroke	Telestroke consultation via videoconferencing improved care in 95% of the cases.
Silva et al (2012) [16]^a^	Cross-sectional survey	Barriers and facilitators	Stroke	Status of telestroke in the United States. The top 3 clinical needs met by telestroke were emergency department consultation (100%), patient triage (83.8%), and inpatient teleconsultation (46.0%).
Kahn et al (2011) [15]^a^	Working group statement	Guidelines	Intensive care	This working group meeting was convened to address methodological and knowledge gaps in the field. It proposed adoption of a common framework to facilitate standardized conduct of telemedicine studies in the ICU^c^.
Audebert et al (2005) [18]	Retrospective	Feasibility	Stroke	Telemedicine provided a cost-effective method to recommend use of thrombolysis among patients presenting with stroke in rural regions.
Lilly et al (2011) [14]^a^	Prospective stepped-wedge clinical trial	Effectiveness	Intensive care	Implementation of a tele-ICU intervention was associated with reduced adjusted odds of mortality and reduced length of hospital stay, as well as with changes in best-practice adherence and lower rates of preventable complications.
May et al (2003) [20]	Qualitative study	Implementation	N/A	Complexity exists at 4 discrete levels in any given telehealth context: implementation, adoption, translation, and stabilization. This complexity is often underestimated, leading to failed scale-ups.
Sood et al (2007) [10]^a^	Literature review	Theoretical underpinnings	N/A	Defined modern telemedicine after a careful review of 104 publications.
Audebert et al 2006 [17]	Prospective	Feasibility	Stroke	The telestroke concept promises better coverage of systemic thrombolysis in nonurban areas.
Audebert et al 2006 [19]^a^	Nonrandomized clinical trial	Trial	Stroke	Treatment in rural hospitals independently reduced the probability of a poor outcome compared with controls.

^a^Purple nodes in Figure 7 representing seminal work in the area of telemedicine.

^b^N/A: not applicable.

^c^ICU: intensive care unit.

### Clusters of Research in Telemedicine From 2015 to 2019

**Figure 8:**
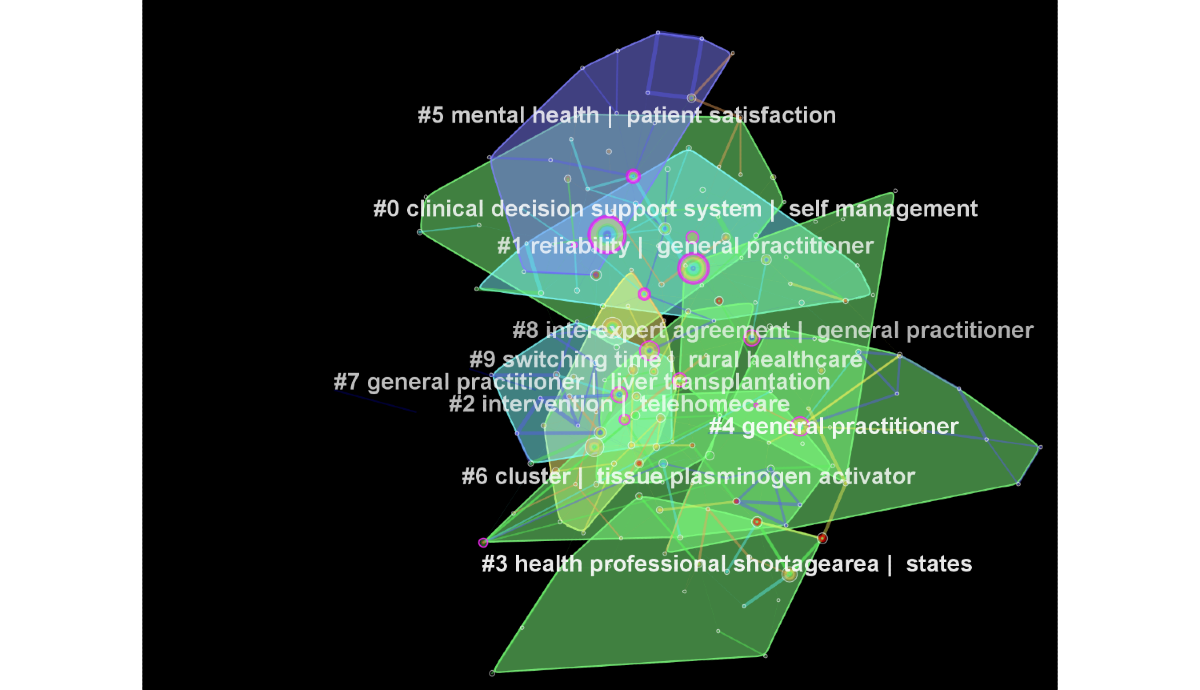
Clusters of research in telemedicine from 2015 to 2019.

From 2015 to 2019, a total of 4493 records were published, which were cited a total of 141,702 times. Cluster analyses yielded a parsimonious cluster network with a modularity of 0.69 and a silhouette value of 0.39. To get a snapshot of research themes in this period, we analyzed a total of 205 nodes with 345 edges. A total of 27 clusters of research in telemedicine were identified, out of which 12 had an acceptable silhouette value. In size, these clusters ranged from 8 to 23 studies, and modularity values ranged from 0.97 to 0.71. These clusters fell into 4 major themes: (1) clinical decision support systems, (2) reliability, (3) access to health care, and (4) medical conditions (Figure 8).

#### Clinical Decision Support Systems

The zeroth cluster pertained to clinical decision support systems to aid in self-management (TF-IDF), explored in the context of ulcerative colitis (LLR) and lung cancer (mutual information).

#### Reliability

A total of 2 clusters (1 and 8) focused on reliability and interexpert agreement (TF-IDF) pertaining to telemedicine-aided diagnoses mediated by general practitioners (TF-IDF), especially in the field of teledermatology (LLR).

#### Access to Health Care

A total of 3 clusters (2, 3, and 9) pertained to this thematic area of telemedicine, defined by access to telemedicine in shortage areas (TF-IDF) to aid in the diagnosis of diabetic retinopathy. Rural health care was an important component of this research theme, where the issue of switching time between pediatric consultations was thoroughly researched.

#### Medical Conditions

A total of 4 clusters (4, 5, 6, and 7) focused on both acute and chronic conditions, for instance, general practitioner–mediated mental health care, especially in PTSD. Cluster 5 pertained to telecardiology, in which heart failure, remote monitoring of pacemaker activity, and patient satisfaction were important areas of research. In accordance with research prior to 2015, acute ischemic stroke and thrombolysis were important research areas in telestroke. In the specialty of tele-ICU, the care of critically ill patients, especially those undergoing liver transplantation, and economic outcome were the most researched areas.

#### End Consumer Research

Patient compliance, safety, and satisfaction were explored in 2 clusters (10 and 11).

### Landmark Publications From 2015 to 2019

This period of scholarly activity in telemedicine continued to be influenced by 4 studies published prior to 2015 [13,14,22-24], pertaining to tele-ICU, telestroke, tele–mental health, and facilitators and barriers to telemedicine. The majority of publications unique to this time period were literature reviews, systematic or otherwise (n=10), followed by retrospective studies (n=2) and a clinical trial (n=1). Major themes in this era were effectiveness and cost-effectiveness research (both primary and secondary). In addition, the connected health model of health care, which governs telemedicine, and the standardized framework for assessment of telemedicine commissioned by the European Commission were deemed central in these collaborative networks.

The most influential review in this period pertained to chronic diseases and was an evidence synthesis report on 141 randomized controlled trials relating to asthma, COPD, diabetes, heart failure, and hypertension [25]. It reported strong evidence of publication bias, with 108 randomized controlled trials reporting positive results and almost none reporting harm [25]. Wade et al [26] presented a systematic review regarding economic analysis of telemedicine and concluded that the delivery of health services by real-time video communication was cost-effective for home care and access to on-call hospital specialists.

Effectiveness research was conducted using both primary interventional and evidence synthesis approaches. For instance, Chaudhry et al [27] conducted a clinical trial and showed that telemonitoring did not improve outcomes among patients hospitalized for heart failure. In their reviews, Ekeland et al [28] and Flodgren et al [29] examined effectiveness of telemedicine in multiple conditions [28,29]; Elbert et al [30] focused on effectiveness and cost-effectiveness for somatic diseases and Hubley et al [31], on psychiatric diseases. Bashshur et al [32] examined 3 tracer diseases (heart failure, stroke, and COPD), which, when treated using telemedicine approaches, showed several markers of improvement, such as reduced hospital admissions and readmissions, length of hospital stay, and emergency department visits. Fierson et al [33] reviewed the currently available literature on telemedicine-based remote digital fundus imaging evaluations for retinopathy of prematurity and outlined pertinent practical and risk management considerations.

Kvedar et al [34] presented a model of care to make telemedicine an important part of the US health care system. He reported that care processes in the United States are insufficient to address the mismatch in supply and demand of health care providers [34]. This review presented connected health as a new care model to improve patient care with telemedicine and telehealth. Kidholm et al [35], after synthesizing evidence from a stakeholders meeting, presented a framework for the assessment of telemedicine with 7 important domains: (1) health problem and description of the application, (2) safety, (3) clinical effectiveness, (4) patient perspectives, (5) economic aspects, (6) organizational aspects, and (7) sociocultural, ethical, and legal aspects. A summary of these publications is provided in Table 3.

**Table 3:** Lessons learned from landmark publications from 2015 to 2019.

Author (year)	Study design	Theme	Disease	Lesson learned
Wootton (2012) [25]^a^	Review	Evidence synthesis	Chronic diseases	This study presents an evidence synthesis report on 141 RCTs^b^ pertaining to asthma, COPD^c^, diabetes, heart failure, and hypertension. There was strong evidence of publication bias, with 108 RCTs reporting positive results and almost none reporting harm.
Wade et al (2010) [26]	Systematic review	Economic analysis	—^d^	Delivery of health services by real-time video communication was cost-effective for home care and access to on-call hospital specialists, showed mixed results for rural service delivery, and was not cost-effective for local delivery of services between hospitals and primary care.
Chaudhry et al (2010) [27]^a^	Clinical trial	Effectiveness	Heart failure	Telemonitoring did not improve outcomes among patients hospitalized for heart failure.
Ekeland et al (2010) [28]^a^	Systematic review of systematic reviews	Effectiveness	—	Out of 80 included systematic reviews, 21 showed that telemedicine was effective, and 18 reported that evidence regarding telemedicine was limited and inconsistent.
Kvedar et al (2014) [34]^a^	Literature review	Model of health care	—	Care processes in the United States are insufficient to address the mismatch in supply and demand of health care providers. This review presented connected health as a new care model to improve patient care with telemedicine and telehealth.
Elbert et al (2014) [30]	Systematic review of systematic reviews	Effectiveness and cost-effectiveness	Somatic diseases	Out of 31 eligible reviews, 7 found eHealth to be clinically effective and cost-effective and 13 found it to be promising, while the rest found the evidence to be limited or inconsistent.
Bashshur et al (2014) [32]^a^	Systematic review	General review	Chronic diseases: heart failure, stroke, and COPD	The 3 diseases, when treated using telemedicine approaches, showed several improvements, such as reduced hospital admissions and readmissions, length of hospital stay, and emergency department visits.
Flodgren et al (2015) [29]^a^	Systematic review and meta-analysis	Effectiveness	Cardiovascular disease, diabetes, respiratory conditions, mental health or substance abuse conditions, conditions requiring a specialist consultation, comorbidities, urogenital conditions, neurological injuries and conditions, gastrointestinal conditions, neonatal conditions requiring specialist care, solid-organ transplantation, and cancer	There was high- to moderate-certainty evidence that there was no significant difference between telemedicine and usual health care in improving all-cause mortality and admissions to the hospital. There was some evidence of improved quality of life, lower HbA_1c_^e^ among patients with diabetes, and decreased LDL^f^ and blood pressure. Participants with different mental health and substance abuse problems reported no differences in the effect of therapy delivered over videoconferencing compared with face-to-face delivery.
Kidholm et al (2012) [35]^a^	Recommendations based on workshops with users and stakeholders of telemedicine, initiated by European Commission	Framework for assessment of telemedicine	—	There are 7 domains in MAST^g^: (1) health problem and description of the application, (2) safety, (3) clinical effectiveness, (4) patient perspectives, (5) economic aspects, (6) organizational aspects, and (7) sociocultural, ethical, and legal aspects.
Schwamm et al (2009) [13]^a^	See Table 2	See Table 2	See Table 2	See Table 2
Lilly et al (2011) [14]^a^	See Table 2	See Table 2	See Table 2	See Table 2
Hilty et al (2013) [23]^a^	Review	Effectiveness	Mental health	This review reported that tele–mental health interventions are effective and improve access to care. More research is required on service models and ethical and cross-cultural aspects of tele–mental health.
Dharmar et al (2013) [22]^a^	Retrospective	Quality improvement	Pediatric critical care	Telemedicine consultations were associated with higher physician-rated quality of care and parent satisfaction.
Sanders et al (2012) [24]	Qualitative	Evaluation and barriers to adoption	Telehealth in general	This qualitative investigation examined barriers to participation and adoption of telehealth among people who withdrew from a UK-based clinical trial on telemedicine.
Fierson et al (2015) [33]	Review	Evaluation for retinopathy of prematurity	Retinopathy of prematurity	This report reviewed the currently available literature on RDFI-TM^h^ evaluations for retinopathy of prematurity and outlined pertinent practical and risk management considerations.
Ashwood et al (2017) [36]	Retrospective	Effectiveness and cost-effectiveness	—	Direct-to-consumer telehealth may increase access to care but does not decrease spending; 12% of direct-to-consumer telehealth visits replaced visits to other providers, and 88% represented new use. Net annual spending on acute respiratory illness increased $45 per telehealth user.
Hubley et al (2016) [31]	Systematic review	Effectiveness	Psychiatric diseases	A large evidence base supported telepsychiatry as a delivery method for mental health services. Future studies will inform optimal approaches to implementing and sustaining telepsychiatry services.

^a^Purple nodes in Figure 7 representing seminal work in the area of telemedicine.

^b^RCTs: randomized controlled trials.

^c^COPD: chronic obstructive pulmonary disease.

^d^Not available.

^e^HbA_1c_: glycated hemoglobin.

^f^LDL: low-density lipoprotein.

^g^MAST: model for assessment of telemedicine.

^h^RDFI-TM: telemedicine-based remote digital fundus imaging.

## Discussion

### Summary

This scientometric analysis presents an overview of scholarly work in the field of telemedicine in the last 10 years. It shows the transition of scholarly work in this field from teleradiology in the previous decade to mental health, stroke, and critical care medicine. Barriers and facilitators to successful implementation of telemedicine were also seen as an important area of research in telemedicine. Collaborative networks between regions and institutions revealed collaborative links between central global institutions and LMICs, showing a transfer of technology and expertise to disparate regions. Among the LMICs, China and India are emerging as big players in telemedicine.

### General Trends and Transcontinental Collaborations

Our analysis revealed a steadily increasing publication output and citation activity in the field of telemedicine, which is in consonance with previous literature [7,9,25,37,38]. In terms of regional output, a bibliometric assessment of literature in telemedicine from 1980 to 2013 showed that the top 5 countries in terms of publication output were the United States, the United Kingdom, Germany, Canada, and Australia, while China ranked tenth [37]. However, we opine that the centrality or influence of a particular entity in their collaborative networks and the volume of innovative work may be better indicators of progress in a field. In this vein, England, France, Belgium, Portugal, the People’s Republic of China, and Greece appeared to hold significant influence worldwide. Similarly, almost all of the top institutions with regard to publication output were from the United States, which reflects previous literature [7,9,25,37,38]. However, only 1 of the United States–based universities was found to be central in its domain. Top institutions were Columbia University, University of Queensland, University of Toronto, and Karolinska Institute.

Several of the top institutions were involved in collaborations with institutions from LMICs, indicating transfer of technology and expertise. This is an important endeavor, as studying the effectiveness and uptake of telemedicine may decrease disparities in these regions. Portugal, for instance, provides a good case study to examine collaborative networks between high-income and low-income countries. A transcultural pediatric telecardiology service has been established in several Portuguese-speaking African countries in collaboration with Portugal-based universities [39]. This program has been highly successful. For instance, in Angola alone, it has performed 32,685 outpatient teleconsultations (1998 to 2016), saving health system costs [39]. Another important endeavor includes echocardiography services through a telecardiology initiative being provided in Tanzania, Malawi, Mali, and Mozambique with a telereporting center in Italy [40]. On October 26, 2017, another impetus for telemedicine research and implementation was provided by a resolution that created the Comunidade dos Países de Língua Portuguesa’s Permanent Working Group on Telemedicine and Telehealth during the fourth Health Ministers Meeting of the Portuguese-Speaking Countries in Brasília [41].

### Transition of Research Themes

We noticed a transition in research themes in telemedicine during these periods. For instance, the WHO reports cited teleradiology services as being most prevalent worldwide. In line with this, Armfield et al [42], using text-mining approaches, reported that during the early period of telemedicine research from 1970 to 1995, teleradiology and telepathology were the most dominant fields, as well as the first fields to adopt telemedicine. In contrast, research trends in a more recent period (2009 to 2013) focused on cost-effectiveness, and the clinical and discipline-specific terms “diabetic” and “stroke” emerged in this period. Our analyses revealed that these themes progressed into the established fields of telecardiology, telestroke, and tele-ICU. Moreover, we also saw a rise in cost-effectiveness as well as implementation and feasibility research, which are very important aspects in the uptake of telemedicine. All the influential studies in our analyses pertained to these themes.

### Research Gaps and Recommendations for Future Work

Despite a continuous rise in scholarly activity in telemedicine, we noticed several gaps in the literature. For instance, all the primary and secondary research central to telemedicine was conducted in the context of high-income countries, including the evidence synthesis approaches pertaining to implementation aspects of telemedicine. In addition, patient confidentiality and ethical perspectives on the use of telemedicine were nonexistent in our analysis. Most of the telemedicine research in LMICs was driven in collaboration with high-income countries. There is a huge gap in needs-based analysis, eHealth literacy, and inclusion of Indigenous end consumers and stakeholders in the design of telemedicine platforms in LMICs.

There were also no research clusters on improving eHealth literacy, especially in the context of use of telemedicine in LMICs. The lack of strengths, weaknesses, opportunities, and threats analyses, particularly in the evaluation of eHealth literacy among physicians, is a big factor in the failure of telemedicine. This was a major contributory factor in the failure of the Réseau en Afrique Francophone pour la Télémédecine (RAFT) telemedicine software platform in Angola, which enjoyed commitment from the Ministry of Health and local stakeholders but was not taken up by the participating physicians [43].

Telemedicine financing is a critical aspect for sustainability and most often not covered in studies. The development of telemedicine on a global scale will require more sophisticated business models. Additionally, telemedicine skills development is very seldom provided by medical schools.

Telemedicine is still in its infancy in LMICs, and there is a lack of clarity in several important aspects, such as the development and adoption of ethical standards, treatment protocols, and guidelines. Medical informaticians should liaise with health care centers, physicians, and medical ethicists to develop software promoting an ethos of confidentiality, privacy, and security during the sharing of sensitive data.

### Conclusion

The findings in this investigation suggest a rapid development in the field of telemedicine, albeit prior to the COVID-19 pandemic. We expect that the research landscape and implementation of telemedicine infrastructure may see exponential progress during and after the COVID-19 period. This is also echoed in the recent report by the American Medical Association, which predicts that “after COVID-19, $250 billion in care could shift to telehealth, boosting research and infrastructural development” [44].

## Abbreviations

COPD: chronic obstructive pulmonary disease
ICT: information and communication technology
ICU: intensive care unit
LLR: log likelihood ratio
LMIC: low- and middle-income country
mHealth: mobile health
PTSD: posttraumatic stress disorder
RAFT: Réseau en Afrique Francophone pour la Télémédecine
TF-IDF: term frequency-inverse document frequency
WHO: World Health Organization
WOS: Web of Science
